# Supplementary material for: Identification of photoperiod-induced specific miRNAs in the adrenal glands of Sunite sheep (Ovis aries)
Source: Front Vet Sci. 2022 Jul 22;9:888207. doi: 10.3389/fvets.2022.888207 (PMC9354845; doi:10.3389/fvets.2022.888207)
Supplement: Supplementary file 1 [file Data_Sheet_1.ZIP › Supplementary file/miRDeep2 file.zip!/miRDeep2 file/Know_Structure/LP42A1/oar-mir-376a.pdf]

miRBase precursor : oar-mir-376a
Total read count : 121
oar-miR-376a-5p read count : 59
oar-miR-376a-3p read count : 57
remaining reads : 5

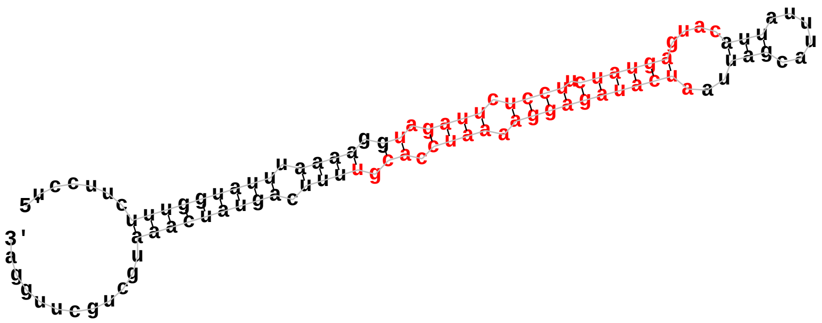

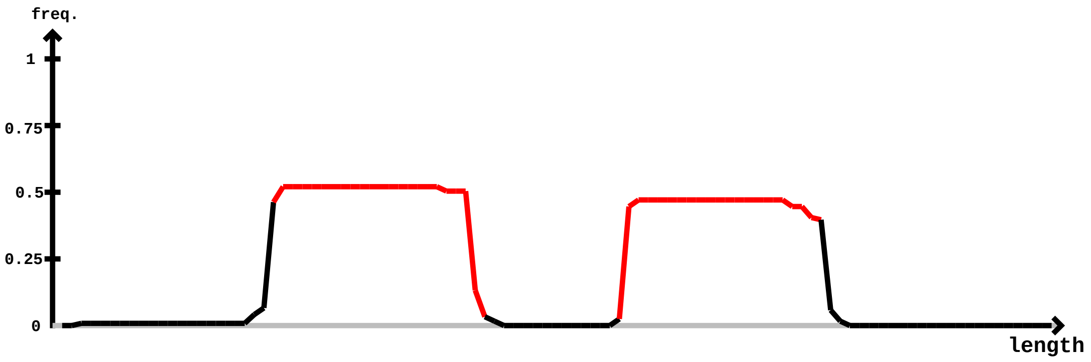

oar-miR-376a-5p

oar-miR-376a-3p

```
5'- uccuucuuugguauuuaaaagguagauucuccuucuaugaguacauuauuuacgauuaaucauagaggaaaauccacguuuucaguaucaaaugcugcuugga  -3'    exp
    ......((((((((.((((.((.((((.((((.((((((....(((......)))...)))))))))).)))).)).)))).))))))))..............    reads    mm    sample
    .ccuucuuugguauuuaaaag..................................................................................    1        0     seq
    ...................agguagauucuccuucuaug................................................................    1        0     seq
    ...................agguagauucuccuucuaugagu.............................................................    3        0     seq
    ....................gguagauucuccuucuaugagu.............................................................    3        0     seq
    .....................guagauucuccuucuaugagu.............................................................    36       0     seq
    .....................guagauucuccuucuaugagua............................................................    9        0     seq
    .....................guagauucuccuucuaugaguac...........................................................    2        0     seq
    .....................guagauucuccuucuaugaguaca..........................................................    2        0     seq
    ......................uagauucuccuucuaug................................................................    1        0     seq
    ......................uagauucuccuucuaugagu.............................................................    3        0     seq
    ......................uagauucuccuucuaugagua............................................................    3        0     seq
    .........................................................aaucauagaggaaaauccacgu........................    3        0     seq
    ..........................................................aucCuagaggaaaaucc............................    1        1     seq
    ..........................................................aucauagaggaaaaucc............................    2        0     seq
    ..........................................................aucauagaggaaaauccac..........................    5        0     seq
    ..........................................................aucauagaggaaaauccacg.........................    1        0     seq
    ..........................................................aucauagaggaaaauccacgu........................    37       0     seq
    ..........................................................aucauagaggaaaauccacguu.......................    5        0     seq
    ...........................................................ucauagaggaaaauccacgu........................    1        0     seq
    ...........................................................ucauagaggaaaauccacguuu......................    2        0     seq
```
